# Ultra-low-cost Mechanical Smartphone Attachment for No-Calibration Blood Pressure Measurement

**DOI:** 10.21203/rs.3.rs-2561946/v1

**Published:** 2023-03-03

**Authors:** Yinan Xuan, Colin Barry, Jessica De Souza, Jessica Wen, Nick Antipa, Alison Moore, Edward J. Wang

**Affiliations:** 1 Electrical and Computer Engineering Department, UC San Diego, 9500 Gilman Dr., La Jolla, 92093, CA, USA.; 2 Department of Medicine, UC San Diego, 9500 Gilman Dr., La Jolla, 92093, CA, USA.

**Keywords:** Blood Pressure, Smartphone, Low-Cost

## Abstract

We propose BPClip, a less than $ 1 USD blood pressure monitor that leverages a plastic clip with a spring-loaded mechanism to enable any smartphone with a flash LED and a camera to measure blood pressure. Unlike prior approaches, our system measured systolic, mean, and diastolic blood pressure using oscillometric measurements that avoid cumbersome per-user calibrations and does not require specialized smartphone models with custom sensors.

## 1 Introduction

Hypertension, defined as systolic blood pressure (SBP) of ≥ 130 mmHg and/or diastolic blood pressure (DBP) ≥ 80 mmHg [1], affects more than 45% of adults in the US with prevalence increasing with age, and is the most common reason for medical office visits and the use of chronic prescription medications [2-4]. Hypertension remains the leading preventable cause of premature death and disability worldwide, killing almost eight million people every year, and is projected to increase by 60% to affect 1.6 billion adults worldwide by 2025[5]. Hypertension, beyond elevating risks of cardiovascular diseases including stroke, heart disease, chronic kidney disease, and end-stage kidney disease, has also been demonstrated to accelerate cognitive decline for middle-aged and older adults, with growing evidence that hypertension is associated with a higher risk of all-cause Mild Cognitive Impairment (MCI) and Non-Amnestic MCI. It is thought that hypertension may cause cognitive impairment through cerebrovascular diseases, such as atherosclerosis of cerebral blood vessels, and damage to the blood-brain barrier.

While high-income countries have stable rates, hypertension prevalence rates are increasing in low- and middle-income countries (LMICs) due to the aging of the population and increases in exposure to lifestyle risk factors including unhealthy diets and lack of physical activity. Control of blood pressure (BP) in these countries continues to be poor, often less than 10%[5, 6]. Migrants from LMICs, especially refugees, are especially vulnerable to poor BP control due to many post-migratory challenges such as navigating new healthcare systems, cultural and language barriers, and low socioeconomic status, which make them more likely to have undetected or uncontrolled BP[7]. Furthermore, diagnosis in LMIC is often late in the disease, with a lack of early preventative care or proactive health care, as hypertension is largely asymptomatic for many years until complications such as advanced heart or kidney disease are present. There is growing research linking perceived stress, a common condition in refugees, to hypertension development and poor management [8]. Besides ethnicity-based hypertension disparities such as in the case of refugees, poor hypertension management during pregnancy in low-resource settings has been a driver of maternal mortality and preterm births due to pre-eclampsia in various LMICs [9]. Home BP monitoring for better BP control is proven to be a cost-effective and vital technique that addresses some of the barriers to accessing care[10] and diagnosing hypertension.

Smartphone-based digital biomarkers leverage the ubiquity of smartphones to improve access and resources for preventative medicine practices far beyond typical clinical resources[11]. Based on the Pew Research Center, as of 2021, 97% of Americans personally own a cell phone and 87% own a smartphone [12]. Also, the widespread adoption and acceptance of mobile phones in LMICs are on the rise at a rapid rate [13]. Leveraging smartphones to measure BP can significantly reduce the burden for those without regular medical access to gain access to early screening and continued monitoring of hypertension on medication, reducing associated cardiovascular and cognitive risks of chronically uncontrolled hypertension.

In an effort to increase accessibility to this vital measurement, we introduce *BPClip*, an ultra-low cost universal smartphone attachment that enables smartphones to measure blood pressure. Our proposed system uses an oscillometric method to measure blood pressure, the same method utilized by typical automated blood pressure monitors. This is consistent with the proposal by the American Medical Association and American Heart Association, which jointly released a statement supporting at-home blood pressure measurements for more reliable monitoring [14]. Importantly, this statement asserts that at-home blood pressure measurements should maintain the same clinical methods of measuring blood pressure using oscillometric methods, barring approaches such as pulse transit time [15], pulse arrival time [16], or deep learning methods that use PPG and demographics [17, 18] to estimate blood pressure, some of which have previously been enabled using smartwatches and smartphone devices. Prior approaches that investigated the use of smartphones for oscillometric measurement have required specific smartphone models with a pressure-sensitive screen [19] or sensorized devices designed as a smartphone case [20].

In comparison, our proposed solution leverages a plastic clip attachment that costs <$1USD that can fit on any smartphone with a flash light emitting diode (LED) and a camera that converts the smartphone into a device that can measure BP using oscillometry. At scale, we estimate the per unit cost to be about 10 cents, a price point orders of magnitude less expensive than any BP monitor. BPClip achieves this by creating a plastic, sensorless force transducer for the smartphone camera using only a spring and a lens-less optical design, reminiscent of a pinhole camera. As the user presses down on the clip, which is placed over the smartphone flash LED and camera, the spring slowly increases the force applied to the digital artery. The pinhole design projects an image onto the smartphone camera that grows bigger as the clip compresses. This projected image encodes two pieces of information. First, the size of the image encodes the applied pressure. Second, the brightness encodes the pulse amplitude through photoplethysmography. By capturing these two measures simultaneously using the camera, the smartphone can measure an oscillogram to calculate both systolic and diastolic BP.

## 2 Results

### 2.1 Concept

**Fig. 1:**
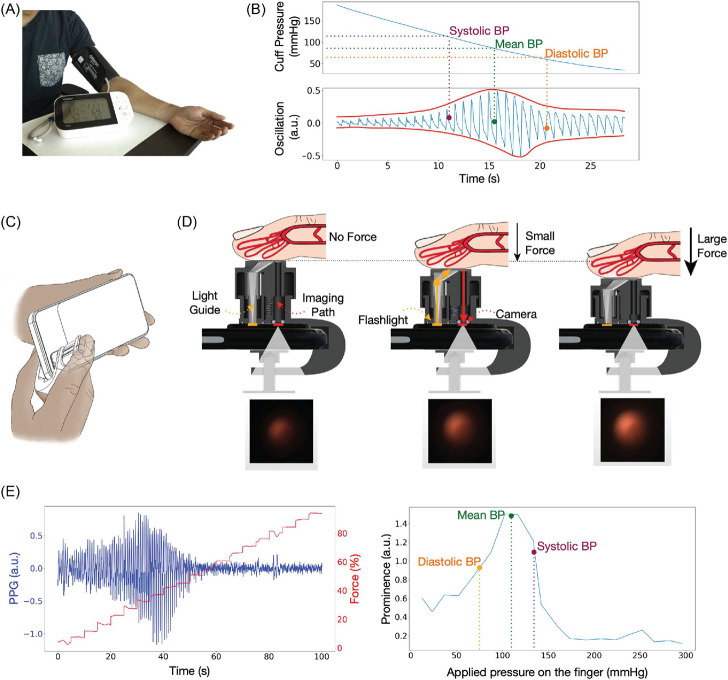
System Overview: Using finger oscillometry to calculate BP. (A) Cuff-based BP monitor (B) Example of an oscillogram obtained from an automated cuff. SBP, MBP and DBP can be calculated from the shape of the envelope of the oscillogram. (C) Using BPClip to measure BP. (D) Light emitted from the smartphone flash travels through the light guide and illuminates the finger. The reflected light travels through the imaging path via a pinhole to reach the camera, forming an image of a red circle. The pulse information is encoded by the brightness of the circle. The pressing force information is encoded by the size of the circle. As the applied force increases, the circle diameter increases. (E) left: pulse and force data extracted from the red circle. right: oscillogram reconstructed from the data on the left.

Similar to traditional automated cuff-based BP monitors, our system utilizes oscillometry to measure BP [21, 22]. As such, our approach does not require per-user calibration. The oscillometric method calculates BP based on the change of blood volume oscillations per heartbeat as the external pressure changes around the artery. Therefore, to use the oscillometric method, a device needs to have three features: 1. the ability to apply pressure on the artery; 2. the ability to sense how much pressure is applied to the artery; 3. the ability to measure blood volume changes as the applied pressure changes. Typically, automated blood pressure monitors exert increasing external pressure on the artery while sensing the pressure and blood volume oscillation with a pressure sensor. (Fig.1A) Fig.1B is an example of the data collected with a typical automated cuff device. The shape of the envelope of the pulse graph makes an oscillogram and is used to calculate BP.

In our smartphone clip-based system, BPClip, we leverage the hardware and computational power that can be found on any modern smartphone to measure BP with the oscillometric principle by mounting a 3D-printed attachment to the smartphone’s camera. To use BPClip, the user holds the phone horizontally with the left hand and pushes down on a spring-loaded clip with the right index finger. (Fig.1C) The user then adjusts the applied force of their finger to a series of varying magnitudes as prompted by a custom smartphone user interface. (Fig.1D) This applies pressure onto the digital artery, specifically the transverse palmar arch artery. Meanwhile, the smartphone flashlight LED illuminates the fingertip by delivering light through a chrome-painted light guide. (Fig.1D) The reflected light travels back through the imaging path via a pinhole, projecting a circular image onto the camera that encodes (1) the pressure applied to the finger and (2) the volume of blood in the finger. (Fig.1D) The pressure applied to the finger is computed from the displacement of the spring-loaded attachment as it is compressed, which is reflected by the size of the circular pinhole projection on the smartphone camera image. (Fig.1D) At higher applied pressure, the pinhole is closer to the camera, creating a larger projection. The blood volume oscillation is detected by the brightness of the pinhole projection. (Fig.1E) When the applied pressure is less than the SBP, the brightness of the pinhole projection fluctuates with respect to the amount of blood through the digital artery, an effect called photoplethysmography (PPG). With more blood, more light is absorbed, resulting in low brightness and a darker projection, and with less blood, less light is absorbed, resulting in higher brightness and a brighter projection. As the applied pressure exceeds the SBP, the blood ceases to flow through the digital artery as the artery is occluded and stops the fluctuation of the pinhole projection. The smartphone application provides visual feedback guiding the user to hold their finger at increasing increments of pre-defined discrete applied forces (Fig.3C), and extracts the pressure and pulse volume by tracking the size and brightness of the projection. (Supplement Video 1)

### 2.2 Prototype

**Fig. 2:**
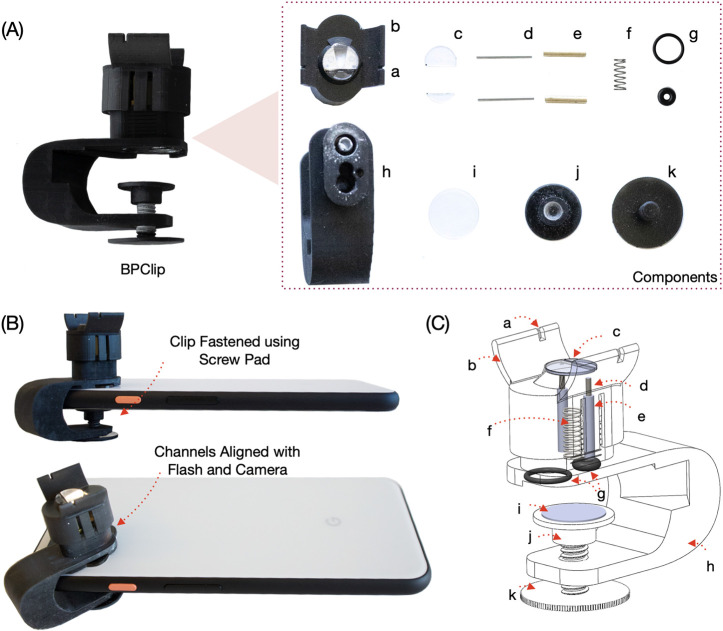
Prototype Component Diagram: Details of the hardware prototype (A) Fully assembled BPClip and disassembled components (B) BPClip mounted on a phone (C) X-ray view of assembled BPClip. Legend: a. notch to align finger b. flange to constrain finger angle c. covers to ensure a flat pressing surface d. rod to ensure smooth pressing e. tube to ensure smooth pressing f. spring g. o-rings to avoid light leakage h. clip base i. anti-slip pad j&k. clamp screw

BPClip consists of a smartphone attachment and an Android application. The 3D-printed attachment consists of a base clip that attaches to the phone, a movable pressing platform that the finger presses, and a compression spring sandwiched in the middle. (Fig.2)

The pressing platform has two flanges that position the finger at the desired angle. On the flange, there is a notch that helps the user align their index finger with respect to the BPClip. On the pressing platform, a 1 cm diameter protrusion is centered over the linear compression spring, which acts as a uniform platform for the finger to press on and apply even pressure.

**Fig. 3:**
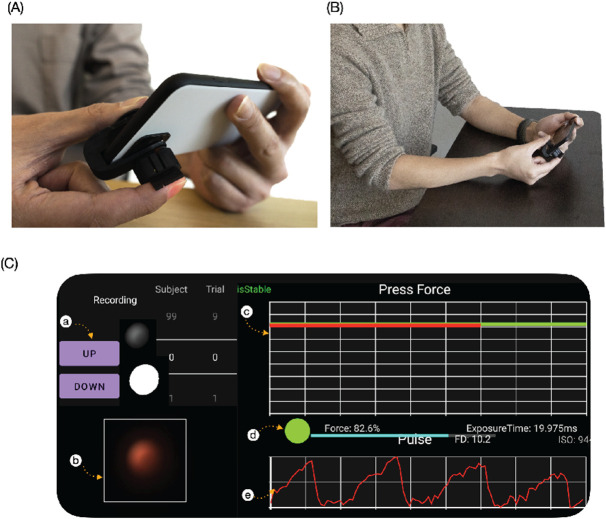
System Usability: Using BPClip (A) The base of the right index fingernail is aligned with the notch. (B) The user holds the phone and clip, with BPClip at heart level. (C) The (user interface) UI layout of the app. Legend: a. button to record force range adjustment b. real-time camera image preview c. Force indicator, red line indicates the current force, green line indicates the target force level d. force range indicator. Red circle indicates the force is out of range. Yellow circle indicates the force is within the range, and the user needs to hold for 2 more seconds to start data recording. Green circle indicates the data is currently being recorded and a 5-second progress bar will show up e. real-time pulse signal

A light guide and an imaging path are designed to guide the light and minimize signal attenuation and noise from light leakage. (Fig.1D) The light guide incorporates a bend to direct the light into the protrusion. This bend accommodates different designs of phones with various camera-to-flashlight distances. In our prototype, we constructed the system to support a 1 cm distance between the camera and the flash LED. To maximize light transfer from the flashlight to the finger, the inner surface of the light guide is coated with chrome paint. The imaging path is right next to the spring and directly under the 1 cm protrusion. Finally, the use of the clip is supported by a custom smartphone application that extracts the PPG signal and applied finger force from the camera in real time. The user interface provides users with visual feedback on the magnitude of force to apply. (Fig.3C)

The base clip is clamped onto the phone with a 3D-printed screw. A silicone pad is placed between the screw and the phone screen to ensure a non-slip interface. To ensure smooth and vertical displacement, two pairs of lubricated rods and tubes are used.

### 2.3 Usage

To use BPClip, the user holds the phone in landscape mode with the left hand, with their right index finger resting on the protrusion and their right thumb resting on the base of the clip. To ensure that the camera can detect PPG signals from the transverse palmar arch artery via the pinhole, the user aligns the base of their fingernail with a notch 2 mm away from the center of the clip, and aligns the length of the finger parallel to the long axis of the clip. (Fig.3A) To eliminate hydrostatic effects, the user holds the phone at heart level, with uncrossed legs, and maintains an upright posture throughout the measurement, similarly to standard protocols utilizing a cuff-based BP measurement (Fig.3B). The user increases the pressure applied to the clip incrementally as guided by the on-screen visual reference. (Fig.3C) At each increment, the user holds the force for 7 seconds, before moving to the next force increment. Data is only recorded during the last 5 seconds of the total 7 seconds to ensure data quality. The application automatically restarts the 7-second measurement if it detects user movement. After 20 levels, the data collection will automatically terminate. This incremental increase serves to subsample the blood pressure oscillogram using the 20 discrete applied forces as points of clean data. (Supplement Video 2)

### 2.4 Cost

A key aspect of our approach is the ultra-low cost nature of the design. Because the mechanism solely relies on a spring for force induction and uses a lens-less optical design to pair with the smartphone, the clip only requires massively manufacturable mechanical parts. Currently, our proposed BPClip system costs under $1USD to produce. The cost consists of the spring ($0.03), the acrylic cover ($0.001), two O-rings ($0.04), the anti-slip pads ($0.03), the metal rod and tube ($0.15) and the resin 3D printed material ($0.55), totaling $0.80. Even at low production, our proposed system is substantially less expensive than any commercial blood pressure monitor. With the majority of the cost from 3D printed plastic, this cost will be substantially lowered at scale with injection molding and bulk component costs.

### 2.5 Accuracy

**Fig. 4:**
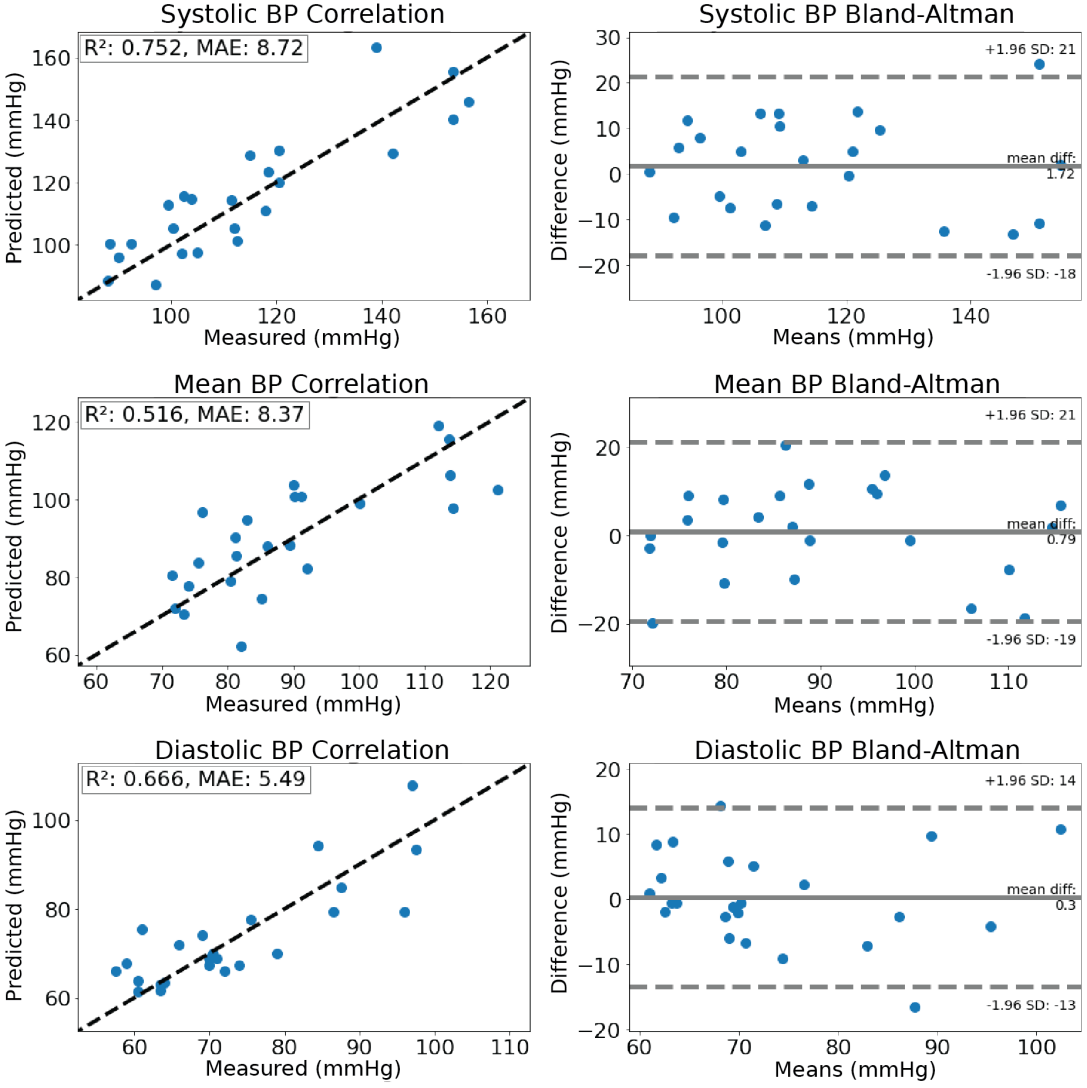
Blood Pressure Estimation Accuracy: Accuracy of blood pressure estimation with Leave-Two-Out Validation (12-fold cross-validation with N=24 subjects) shown in correlation and Bland-Altman plots. The dashed lines in correlation plots represent the best-fit line, where the measured value equals the predicted value.

The accuracy of BPClip is validated on data collected from 24 users with SBP ranging from 80-156 mmHg and DBP ranging from 57-97 mmHg. Figure 4 shows the correlation and Bland-Altman plots for the systolic, mean, and diastolic BP measurements. The systolic and diastolic reference measurements were recorded using a standard arm cuff device. The mean BP reference is calculated from the reference measurements as *meanBP*(*MBP*) = 1/3 *SBP* + 2/3 *DBP* [23]. BPClip’s measurement achieved a mean absolute error (MAE) of 8.7±10.0, 8.4±10.3, and 5.5±7.0 mmHg and bias of 1.72, 0.79, and 0.3 mmHg for systolic, mean, and diastolic BP, respectively. Five subjects were excluded from analysis for the estimation accuracy analysis due to either poor perfusion which resulted in the inability of the system to acquire pulse data (N=4), or an atypically large difference between SBP and DBP of greater than 80 mmHg (N=1).

### 2.6 Usability

Because using BPClip requires the user’s active interaction, we analyzed how much time it takes for a user to complete one measurement. The time spent in each trial was calculated from metadata for each participant. The minimum possible time for a user to complete a measurement is approximately 140 seconds for the measurement, given 7 seconds per level for 20 levels if the user completes each level without repeating.

On average, it took 251±127 seconds for the first trial and 212±45 seconds for the second trial. Because the right index finger is used for BPClip, we also recorded if the participant is left-handed or not. Among the recruited participants, 6 of them were left-handed, but none of them reported difficulty using BPClip. A t-test suggested that the difference in time to complete one measurement trial is not significant between left-handed users (N=8) and right-handed users (N=21). (p-value=0.85)

## 3 Methods

### 3.1 Study Design

29 study participants were recruited from UC San Diego main campus and UC San Diego Medical Center. (Details in Appendix A, B) (SBP: 116.8±20.3mmHg, ≤ 110mmHg: N=12, 110 to 130mmHg: N=10, ≥ 130mmHg: N=7; DBP: 73.5 ± 12.3mmHg, ≤ 70mmHg: N=12, 70 to 80mmHg: N=9, ≥ 80mmHg: N=8) Informed consent was obtained from all participants. All the methods included in this study are approved by the University of California, San Diego Institutional Review Board (IRB) (IRB Approval #804668), and all methods included in our study are in accordance with the relevant guidelines. After being recruited for the study, participants were asked to warm their hands with heat packs to promote blood flow to the fingertips. The participants then received instructions on how to use the device and performed a practice measurement using BPClip. During the practice measurement and all subsequent measurements, the participant was positioned in a seated position with their hand resting on a table surface at the level of their heart, such that both the left bicep and right index finger are at the level of the heart. The participants then performed 2 consecutive measurements with BPClip. Immediately before and after BPClip measurement, an automated blood pressure cuff device [Omron, model: BP7350] was used to measure BP on the left arm. The average of the two cuff measurements was used as the reference.

### 3.2 Prototype: Hardware

BPClip is designed with SolidWorks CAD software and printed with a resin 3D printer (Any-cubic Photon Mono X with Hard Tough Resin - Black). The clip dimensions are 5.1x4.1x2.3 mm. The clip is clamped onto the smartphone (Pixel4, Google), with a 3D-printed screw. The clip consists of 3 main components: the base clip, the pressing platform, and the compression spring.

The base clip is a 3D-printed clip-shaped piece that is mounted to the smartphone. It also includes a 3D-printed screw and nut which can tighten to help fix the clip in position. To ensure a firm and non-slip interface, a silicone pad is used to increase the friction between the clip and the phone screen. The bottom piece is flush with the camera and flashlight LED.

The pressing platform is also 3D-printed and wraps around the bottom piece. It comes in contact with the user’s finger and is displaced as the user applies force to it. The contact area is a circular protrusion with a diameter of 10 mm. To ensure an even surface for the finger contact area, clear acrylic pieces are placed over the openings of the illumination slot and pinhole. Two flanges are used to constrain the angle of the index finger. Two notches are used to help users align their index finger with the pinhole.

Interfacing the top and bottom is a compression spring with a k-constant of 0.49 N/mm, placed in between the pressing platform and base clip. It renders the pressing platform the ability to move up and down, depending on the amount of force applied by the finger. The spring is pre-loaded with a force of 0.1 N. 6.7 mm more space is allowed for the spring to compress, rendering the potential range of force applied to the finger from 0.1 N to 3.3 N. With an area of a circle of 10mm diameter, the pressure would range from 9.5 mmHg to 315 mmHg. Because the prototype is constructed with 3D printing, surface finishes are not perfectly smooth compared to injection molded plastic parts. Two pairs of guiding rods and tubes, with lubricants applied, are added to the pressing platform and base clip respectively to allow for smooth uniform displacement.

A light guide and an imaging path are designed to maximize the light transfer and minimize light leakage. The light guides direct the flash into the protrusion and into the finger. To maximize light transfer from the flash to the finger, chrome spray paint is applied to the inner surface of the light guide. A pinhole projects the light from the finger onto the camera via the imaging path. At the end of the light guide and imaging path, two O-Rings were respectively padded in between the bottom piece and the phone to reduce the external light influence.

### 3.3 Prototype: Software

We developed an Android smartphone application to be used with BPClip. The application has 3 main features. First, it infers the applied finger force from the size of the pinhole reflected on the image captured by the camera. The pinhole is projected to the camera sensor as a circle. For each frame, the area of the circles is calculated from the number of pixels with a value above 20 (the image is encoded with unsigned 8-bit integers). To account for different baseline values within the circle due to different amounts of blood circulation, a histogram equalization operation was applied to the raw image before area calculation. The diameter of the circle is then calculated from the area of the circle. The force is plotted in real-time in the force plot UI. To avoid unnecessary confusion for the user, the force signal was processed with a low pass filter to remove the PPG artifact before being plotted.

Second, the application can extract the PPG signal from the pixel values within the circle. For each frame, the average value of the pixels within the circle that have a value between 20 and 254 is calculated to be the PPG signal. The PPG is plotted in real-time in the PPG plot UI.

Third, the application has a UI that helps the user maintain the applied finger force at a specific level. This UI consists of a brightness adjustment feature and a feedback feature. Because the brightness depends on the amount of total blood circulation as well as the skin tone, the size of the circle projected onto the camera at each force level changes depending on the person. To adjust for this, a simple brightness adjustment is performed before each measurement. To perform an adjustment, at the beginning of a measurement round, the circle size when the user rests the finger on the clip with no force is recorded (Force Scale 0%), as well as the circle size when the clip is completely pressed down. (Force Scale 100%). By capturing the 0% and 100% circle sizes, the ensuing measurement can be performed in proportion. 20 force levels are then equally divided from 5% to 95%. During the trial, a green line representing the current target force level appears in the force plot. An additional red indicator line showing the current force applied helps guide the user to adjust the applied force to meet the target force. While the applied finger force does not yet match the target, the indicator line remains red. As soon as the force is in the range close to the target (±2%), the indicator turns yellow. The user is then expected to hold their finger in position to maintain a constant applied force. If the force stays within the range, after 2 seconds the indicator turns green from yellow, where the data is collected for 5 seconds. If the force goes out of the range (±2%) anytime when the indicator is yellow or green, the indicator changes back to red, and any data collected will be discarded. The data for the current force level then needs to be recollected.

### 3.4 Data Preprocessing

This section elaborates on the process of preprocessing raw data recorded on the smartphone. As thoroughly explained in the prototype hardware section, the camera images contain a pinhole projection. The diameter of the pinhole projection corresponds to the force exerted on the finger. The pixel intensity fluctuations of the pinhole projection correspond to blood flow through the palmar arch artery.

For the data analysis, we only use data captured during the 5-second intervals at the specified force levels from the smartphone application. For the applied force, this smartphone application ensures ±2% tolerance on each force level. At each of the 20 force levels, the 5 seconds of recording allows for approximately 3-7 complete pulses of blood flow, as measured by the pixel intensity of the pinhole projection. For each of these force levels, we applied a 0.5 to 10 Hz band pass filter and then averaged the peak-to-trough amplitude of all complete pulses using peak prominence. This results in one average peak prominence value for each of the 20 force levels. These 20 points make up the subsampled blood volume oscillogram used to calculate blood pressure.

During the data preprocessing, we excluded participants for the following reasons:

- The participant did not have sufficient perfusion to create a PPG signal with significant enough prominence values for interpretation. (N=4)
- The participant had abnormal blood pressure with more than 80mmHg difference between the reference systolic and diastolic BP measurements. (N=1)

This resulted in N=24 participants for the data analysis and validation steps of the study.

### 3.5 Data Analysis

This section describes our process of using pre-processed data to predict blood pressure. In estimating systolic and mean BP from the 20-point blood volume oscillogram, we exclude the lowest and highest force levels, because when at the lowest force, the finger can sometimes leave the clip, and at the highest force, the spring is fully compressed and the applied finger force may exceed the spring load. From the remaining 18 oscillogram points, we normalize the points between 1 and 0. We use the 18 oscillogram points as the input into a Least Absolute Shrinkage and Selection Operator (LASSO) Regression[24] to estimate systolic and mean BP. For estimating diastolic BP, we use a linear regression model, where the only input features are the predicted systolic and mean BP values. For all BP prediction validation results, we use a leave-two-out validation (12-fold cross-validation) with N=24 participants.

### 3.6 Data Availability Statement

The data that support the findings of this study are available from the corresponding author upon reasonable request.

### 3.7 Code Availability Statement

The code that supports the findings of this study is available from the corresponding author upon reasonable request.

## 4 Discussion

A key finding in our work is that there is no need to perform a per-user or per-device calibration. This is enabled by the simple top-bottom brightness adjustment that is included at the beginning of a measurement. By asking the user to hold the clip at the furthest compression and then once again at the closest compression, our system adjusts for the total brightness as reflected by a person’s finger. Thus, no matter the skin tone (where darker would absorb more light) or with a dimmer flash (as not all phones have the same brightness), the brightness adjustment equalizes the proportional amplitude of the PPG accordingly.

As with all optical-based pulse sensing systems, skin tone needs to be considered. Although a formal skin tone study was not performed, our approach does not have a fundamental limitation due to skin tone beyond signal strength. Darker skin tones can absorb more light, thus leading to less total light reflected. However, unlike calculating oxygen saturation (SpO2), our method does not depend on the distribution of absorbed light at different wavelengths, but rather only depends on the total amplitude of the signal. Our current prototype addresses this sufficiently through chrome-painting the light guide from the flash, which channels a significant amount of light from the phone flash. Additionally, for darker skin tones, we can increase the sensitivity of the camera without compromising the sampling rate. Increasing the exposure time, however, is not a good solution because that would interfere with the sampling rate of the pulse signal.

More importantly, our method requires adequate blood circulation in the finger to capture a strong pulse signal. To ensure an adequate amount of blood circulation in the finger, we provided a hand warmer for participants to warm their hands prior to measurement. Additionally, the participants’ perfusion index (PI) was checked using a pulse oximeter [NuvoMed, A310] before the data collection. If the PI ≤ 1%, the participants continue to warm their hands to raise the PI to above 1%.

A key contribution of our work is that the design of this ultra-low cost blood pressure monitoring attachment is suitable for almost all smartphones on the market. The studies presented in this paper were conducted using the Google Pixel 4 smartphone, however, because BPClip only relies on the smartphone camera and flashlight, our approach is suitable for almost all phones. BPClip can be expanded to other smartphone models with minimal changes, as most phone models would only require changes in the distance between the flashlight and the camera. Our current design already takes that into account and uses a bend in the light guide to bring the flashlight to the finger from various distances.

A limitation of our system, compared to a fully automated cuff is that the user needs to have a reasonable level of finger dexterity to be able to use BPClip as the user needs to be able to hold the index finger steadily while exerting various amounts of force. Therefore, for some older adults with weakened grip and/or hand tremors, it can be difficult to use BPClip. However, we find that our middle-aged participants had no trouble using the device.

## 5 Conclusion

Health monitoring should be available to everyone, no matter their country of origin, their skin tone, their sex, and most of all, their income. In this paper, we propose a solution to democratize blood pressure (BP) monitoring by converting the billions of smartphone cameras, even the cheapest ones, into BP monitors with an ultra-low-cost plastic clip that can be produced at-scale for mere cents. Our current prototype costs less than $1USD to manufacture at low quantities, but at scale, we expect that a blood pressure monitor will reach the cost of less than a US quarter. So cheap, in fact, our goal is that BP monitors should be provided to anyone who needs them, much like the way a pack of floss is given out at dentists or stress balls at fairs.
